# Topics of Public Interest

**Published:** 1918-11

**Authors:** 


### TOPICS OF PUBLIC INTEREST

**Major General Wm. C. Gorgas,** Surgeon General of the Army, was retired at the age limit, Oct. 4. He was at the time in France and will probably be retained in an advisory capacity. He is succeeded by Major General Merritte W. Ireland, formerly acting as the representative of the Surgeon General for the American Expeditionary Force. The place of the latter will be filled by Brigadier General Robert E. Noble.

**British Professional Statistics.** The United Kingdom has 43,819 physicians and 5,500 dentists. Of the former, England and Wales have 23,175; Scotland 13,918; Ireland 6,176. The annual increase has been from 501 between 1912 and 1913 to 338 between 1916 and 1917. Deaths, authenticated, have been 746, 1068, 800, 1005, and 831 1913-1917, respectively. The number of students in 1917 was 7600, 1500 more than in 1916 but less than the ante-bellum average. Whether these figures include men in military service and military deaths or not, is not stated.

**German Man Power** now totals about 2 million on the western front, according to count of divisions. The losses in prisoners alone have been stated to be 185,000 for Aug. and Sept. and the definitive losses may be estimated at about three times this number or a rate of about one division a day, according to the present reduced make-up of a division, from the original of about 13,500 to 10,000. Sept. 10, the number of divisions on the western front was stated to be 195. It is also said that the class of 1920 has been withdrawn from active service at the front as being too young (17) and poorly nourished to stand the strain. As previously explained, the uprising generation cannot be depended on for any nation at war, with its maximum militia engaged, to do more than make good the ordinary wear and tear aside from military casualties, and Germany has already drawn on the future to the extent of two annual classes. If this rate of attrition can be kept up, it is a comparatively simple problem to estimate Germany's endurance, providing that forces are not available from other fronts or from unforeseen sources. The present force is a little short of the estimate of 5,000 men per mile considered necessary, including exchange troops and immediately available reserves. The time seems to be near when total casualties will have almost as great an effect as definitive casualties, which equal 40-50% of total. Then, a shortening of front will become imperative.

---

**State Commissioner Biggs** states that bacteriological research on cases of this disease point to the probability that it is caused not by any new organism, which would justify the use of the word "Spanish" as describing it, but to the so-called Pfeiffer bacillus or influenza bacillus which caused the serious world epidemic from 1889 to 1892.

A definite conclusion as to the causative factor cannot, however, be reached until further laboratory investigations have been completed; this the State laboratory, together with many other laboratories, is undertaking at the present time. Clinical observations made a few days ago by a member of the Staff of the State Department of Health on a number of cases occurring in United States sailors showed that the typical form of the disease shows very uniform symptoms. The onset is accompanied by fever which varies from 101 to 105, headache, pains in the muscles and bones, and a good deal of prostration; there is a dry, nagging cough, with at first little expectoration. There may or may not be profuse discharge from the eyes and nose; the throat may be quite red, sometimes intensely so, more often it is not markedly affected. The course of the disease when uncomplicated is short, the temperature falling in from 48 hours to three days to normal. In some cases the disease begins with vomiting and diarrhea and the catarrhal symptoms only appear later.

In addition to typical cases, many atypical ones occur which present only the symptoms of an ordinary cold with no marked rise in temperature or great prostration, and even in the same household will be found typical and atypical cases, in all probability caused by the same type of organism, the different reaction being dependent upon the different degrees of resistance of the individuals affected.

The disease is seldom fatal of itself except in very susceptible or weak individuals. The complications, however, notably pneumonia, are very much to be dreaded; many deaths have already occurred from pneumonia.

Commissioner Biggs stated that strict quarantine measures, such as one would take against other infectious diseases, while theoretically desirable, are not practical in view of the highly contagious character and the wide spread extent of the disease, and the general susceptibility to it.

Like other infections of the respiratory tract, influenza spreads from one individual to another by contact with the discharges from the nose and throat, scattered by coughing and sneezing, the use of common eating utensils, towels, etc. For this reason, insofar as possible badly aired and crowded places should be avoided, as well as excesses in eating or drinking, or any over exertion or over fatigue, which tend to weaken the bodily resistance.

There is no known cure for the disease.

---

**American Doctors** are enrolling in the Volunteer Medical Service Corps at a gratifying rate. 19,225 applications have been received to date. In response to the appeal that State executive committees be enlarged and strengthened for the work of handling the Volunteer Medical Service Corps matters, and also the request that a representative in each county be quickly designated, practically all of the States in the country have complied and have sent in the personnel of the State executive committees and of the county representatives to the headquarters of the Volunteer Medical Service Corps in Washington. The make-up of each state committee has been the joint work of the central governing board and the State committees, medical section, Council of National Defense, in each State. There has been the closest co-operation, and the officials of the central governing board are assured that the executive committees will all hold frequent meetings and keep in close touch with the situation in their respective States.

---

**Whale Meat,** long in use in Japan, has assumed an importance for all countries with an available supply, even occasionally, as a substitute for ordinary meats. It is described as palatable and resembling beef, being coarser in texture and darker, but not tough. It has sold at 10 cents a pound in coast cities.

---

**Physical Tests for Airmen are Novel and Exciting..** All men who have won their wings in the United States air service are now required to pass a new heart, lung, ear, and eye test to establish their physical and mental fitness when high in the air and particularly to indicate at what heights they are in a condition to fly. Cadets receive a test before they finish their schooling; flyers are given these tests periodically to eliminate any whose physical or mental efficiency has become in any way impaired.

To stay in the rarified air at an elevation of 20,000 feet for any length of time has been found to be a strain on even the most physically perfect. It has also been discovered that many of the most seasoned fliers can not undergo the sudden quick changes in altitude occasioned by diving and climbing without physical deterioration. It was recognized as too great a risk to subject these men to actual flying tests. So the medical laboratory at Hazelhurst field undertook to devise some way of getting the same results by means of a ground test.

In the early tests the pilot was placed in a steel airtight cylinder from which the air was gradually exhausted and then replaced, to simulate a flight into the rarified air of high altitudes and back to earth, but today the pilot sits comfortably in the same room with his examiners. His nose is clamped so that he can not breathe through it. Over his mouth is placed the breathing apparatus, which is connected by tubes with a tank of measured air, and with instruments that record every breath he takes. The air is analyzed at various stages of the run. As fast as he exhales the air is taken into a reservoir where it is cleared of carbon dioxide, and then returned to the tank. Gradually he uses up the oxygen and thus air conditions of high altitudes are duplicated. The higher one goes up, the rarer the air becomes; just so with the man under test.

The man under test is kept fairly busy, just as he would be piloting a plane. Before him on a table is a bank of small electric lights, one or another of which flashes every 5 seconds. These he must extinguish as fast as he observes them and before they go out. He has but a few seconds. Below the lamps is a corresponding set of buttons which, when touched with a pointer held in the right hand, extinguishes the respective lights. Two observers watch him constantly and check his errors or delayed actions.

Another instrument before him is an ammeter which acts similar to a speed dial on a plane, and, accordingly, in the test must be kept at a constant point.

As time goes on (and the test lasts for about 30 minutes) the pilot becomes a bit groggy or sleepy from lack of oxygen, just as he would at the corresponding altitude, and this condition becomes manifest in changes in the action of his heart, eyes, ears, and brain.

A few minutes after his release from the apparatus all signs of his recent fatigue pass away and he becomes normal again.

---

**Italy's Total Military Force** has amounted to 5 million, with definitive losses to date of about 1,350,000.

---

**Army Death Rate for Disease Only 2.18 per 1,000 Annually.** American troops both here and overseas continue to establish good health records. For the two months' period ending August 31 the combined reports of the American Expeditionary Forces and all troops stationed in the United States show an annual death rate for disease of 2.18 per 1,000—a fraction more than 2 men per 1,000 per year. The annual death rate for disease of men of military age in civil life is 6.7 per 1,000. The combined reports show that generally the health of the soldiers overseas is better than that shown by the men in training in this country. This is largely due to the fact that only men in the best physical condition are being sent to France, with the result that the reports of the troops in America carry the men unfit for overseas duty.

---

**Women Doctors** are urged to join the Volunteer Medical Service Corps. We have heard so much about the splendid work which has been done by the group of English physicians known as the Scottish Women's Hospitals. Let us show how quickly and ably the American woman doctor can respond to this call of help.

---

**Women Physicians** who are qualified for anesthetic service or who are competent to be intensively trained are requested to get in touch, at once, with Dr. F. H. McMechan, Sec'y Interstate Anesthetists, American Anesthetists, Avon Lake, Ohio.

---

**Military Training and Post Bellum Industry.** The question has arisen whether military discipline will fit or unfit for various industries in peace, whether subordinate positions will crush out initiative and higher positions spoil the individual for subordinate civil work or whether the reaction will lead too many men to seek executive and independent work. So far as direct training is concerned, no soldier will benefit more than the medical officer. For instance, a personal experience frequently exceeding 100 cases a day and including some thousands of full physical examinations cannot make one a better physician. Can some of our subscribers discuss this subject from actual observation after the Civil War?

**Results of Reduction of Alcoholic Consumption in Great Britain.** The weekly average of arrests for drunkenness declined from 672 in 1913 to 147 in 1917; delirium tremens cases in representative areas from 143 to 28; deaths from alcoholism from 719 to 222; attempts at suicide from 968 to 452. The last seems to show that the argument that alcohol affords relief from mental suffering is not well grounded.

**The Definitive Losses of the Canadian Army** were 115,806 up to Aug. 1. This total includes a comparatively small number of officers and non-commissioned officers transferred to the British Army. It represents a total depletion of about 20% in 4 years. As troops were sent in gradually, it is not possible to calculate anything like an accurate risk for a definite period but it justifies the belief that the German losses estimated at over 50% of the original militia strength are not exaggerated.

**Butter.** A Federal commission has been appointed to consider butter prices. It is feared that the present high prices will lead to such extensive use of substitutes as to permanently reduce the demand for natural butter, with an ultimate effect on the whole dairy industry. In this connection, it may be well to emphasize the fact that demand and supply are not so closely related as is usually believed. A very rare article may have almost no commercial value. Of course, the selling price of an article in common use does correspond to the abundance but only temporarily. Ultimately the price of anything corresponds not to the demand of the purchaser but to the demand of the producer or distributor for payment for his labor, subject to the opportunity that he has to turn his labor into other more profitable channels.

**Cereals.** The most recent estimates give the U. S. a wheat crop of about 920 million bushels; corn 2,777; oats 1,535. Last year's results were: wheat 650; corn 3,159; oats 1,587. While the total by bushels is about 150 million less than last year, the greater weight, not to mention its superior food value, of wheat, more than compensates. The wheat represents nearly double the amount that would be demanded in peace for our domestic use and at least three times the amount necessary to maintain an ideal state of nutrition of our own people, with a reasonable reliance on other cereals. Personally, we do not regard the mixture of wheat with other cereals or the substitution of corn breads, etc., as at all undesirable. Others may not agree but we have never encountered so wide a variety and such a degree of palatability of bread stuffs as since our sacrifice of wheat began. One item in regard to wheat conservation has scarcely received the attention it deserved. The fact that American troops in Europe could eat white bread and give it to German prisoners, produced a tremendous impression of power. The fact was given wide publicity and cannot fail to penetrate to Germany through neutral countries. It is stated that some German prisoners abandoned hope of victory on this account alone and it is probably no exaggeration that our sacrifice in the use of wheat—really no sacrifice at all—will count for as much in the determination of victory as a division of troops—perhaps more.

---

**Milk.** The Buffalo Common Council has approved an ordinance requiring all milk to be pasteurized except certified raw milk. 90% of all milk sold is already pasteurized largely because pasteurized milk is less perishable than raw. The wholesale price of milk has been set at 32.6 cents per gallon, the retail price is 15.5 cents per quart for Grade A, 14.5 for Grade B.

---

**Paper Shortage.** All periodicals are to some degree limited as to amount and quality of paper. One of our lay contemporaries complains as to the very liberal use of paper by various branches of the government and affiliated commissions in distributing news matter and propaganda with the request for publication.

---

**Weather and Respiratory Infections.** An impartial view of military experience in the last 18 months corroborates, in a practical sense, the old idea of taking cold. The etiologic relation of bacteria to coryza and deeper colds of the respiratory tract cannot be denied, while individual cases show that the climatic element is not absolutely necessary. But so far as epidemics and endemics are concerned, the importance of cold, damp and fluctuations of humidity and temperature within certain limits—and just such limits as the old practitioners had established empirically—cannot be denied. This is true not only of non-specific infections—though the line between non-specific and specific is becoming increasingly difficult to determine—but of unquestionably specific infections like influenza and pneumonia, of such as diphtheria whose relation to taking cold is less plain, and even of measles which is only symptomatically a respiratory disease in the minds of most persons. On the other hand, extreme cold weather, especially with fairly steady temperature and low humidity does not favor the occurrence of respiratory infections nor should one choose warmth and contaminated air in the treatment of an existing case or in the prophylaxis against spread of infection, against free ventilation and cold air.

---

**The Passing of the M. R. C.** The unification of the U. S. Army was a valuable measure, not merely to establish a solidarity of military power but to eliminate a considerable loss of efficiency in the inevitable paper work. It applies, of course, to the medical as to all other departments so that surgeons recently and temporarily from civil practice are now designated M. C. exactly like the regular army surgeons. However, one cannot refrain from experiencing a little sentimental regret at the removal of the distinctive designation of the men whose life work was with the army and whose seniority over volunteer and national guard surgeons was thus indicated. Then, again, many of the M. R. C. men were equally proud of their prestige as civilian practitioners and of their status as volunteers for temporary patriotic duty.

---

**Conservation of Shells and Fruit Pits.** If it had been realized even a year ago that charcoal derived from these substances was of superior grade for gas masks, a large stock could have been accumulated. Looking ahead it may be pointed out that practically all seeds, including the soft contents of pits of fruits, contain proteid to the amount of about 10% and that all seeds contained in dense walls also usually contain large amount of fat—as much as 60% for certain nuts. While certain pits contain cyanogen in combination and others various bitter principles, there is no question but that any objectionable substances can be removed even by such simple domestic procedures as boiling or roasting. Thus there is a considerable potential stock of nutriment that can be utilized if necessary.

